# Assessing Comfort in Urban Public Spaces: A Structural Equation Model Involving Environmental Attitude and Perception

**DOI:** 10.3390/ijerph18031287

**Published:** 2021-02-01

**Authors:** You Peng, Zhikai Peng, Tao Feng, Chixing Zhong, Wei Wang

**Affiliations:** 1 Urban Planning and Transportation Group, Department of the Built Environment, Eindhoven University of Technology, P.O. Box 513, 5600MB Eindhoven, The Netherlands; y.peng@tue.nl (Y.P.); t.feng@tue.nl (T.F.); 2 Department of Architecture, University of Cambridge, 1-5 Scroope Terrace, Cambridge CB2 1PX, UK; zp254@cam.ac.uk; 3 School of Architecture, Hunan University, Changsha 410082, China; zhongchixing@yeah.net

**Keywords:** outdoor comfort, urban public space, environmental attitude, environmental perception, structural equation model

## Abstract

The research of comfort in urban public spaces has become increasingly important for improving environmental quality and encouraging people spend more time in outdoor activities. Among numerous approaches to understand comfort perception, the rational indices based on heat balance theory have prevailed to guide the research and practice in urban planning, design, and management. The limitations of a solely rational index-based approach reveal the necessity for a more comprehensive understanding of comfort by considering a wider range of influential factors from both individual and environmental perspectives during the assessing process. This study conceptualizes individuals’ comfort in urban public spaces as a latent construct, which is measured by indicators regarding perceptions on multifarious meteorological variables. The conceptual framework has been introduced involving hypothetical relationships among individuals’ comfort, attitudes, and environmental perceptions in urban public spaces. A series of field work including microclimate measurements and questionnaire-based surveys were carried out in two public squares in Changsha, China. Based on the dataset derived from 372 questionnaires and related meteorological measurements, this paper examines the relationships between the physical microclimatic variables, individuals’ socio-demographical characteristics and environmental attitudes and perceptions, and outdoor comfort assessment. The estimation results of the structural equation model quantitatively verified the conceptual framework at large, as many hypothetical relationships are identified, which indicates the importance of individuals’ role and the psychological factors in modeling comfort perception. This approach improves the understanding of comfort assessment, contributes to improving the quality of urban environment and the practices of urban planning and management.

## 1. Introduction

A growing concern with population inflation and urban expansion along with the heat island intensification, the lack of ventilation and decrease of greenspace in highly densified built up areas has resulted in deterioration of many climate-induced urban problems [1,2]. Still, the rapid urbanization is expected to emerge globally in the next a few decades which will lead to more and more people becoming urban inhabitants [3], and inevitably cause the expanding demand for more living and working spaces. Meanwhile, the general trend of climate change is increasing pressures to the urban environments and posing environmental challenges to the urban planning for the outcome of public health and sustainable development. In addition, the awareness of the importance of urban bioclimatic conditions is growing along with the demand in the resilient and climate-responsive design for comfortable outdoor open spaces [4,5,6,7,8,9].

In contemporary society, urban inhabitants only spend less than 20% of their time out of buildings in some developed countries [10]. It has long been recognized that comfortable outdoor public spaces meeting the expectations of occupants can attract people to spend more time in outdoor environments and substantially affect the likelihood of archiving sustainable urban development and enhancing the inhabitants’ quality of life [11,12,13,14,15]. As the essential components in urban system, outdoor and semi-outdoor environments can provide open spaces for hosting various recreational, social and commercial activities [16]. On the other hand, the outdoor green spaces in urban areas are facilitating to adapt to the increased heat stress and evolving into an important contribution to the energy efficiency of the surrounding buildings [17,18].

Since the last two decades, the research interest in assessing the outdoor thermal comfort in urban environments has drawn a great deal of worldwide attentions [19]. From the literatures, rapid growth has come at the amounts of studies carried out in different geographical regions with distinct climates for developing and calibrating the model of comfort in outdoor urban spaces [20,21]. The rational indices, such as Physiological Equivalent Temperature (PET) [22] and Universal Thermal Climate Index (UTCI) [23,24], have been widely applied for the comfort modeling and estimation of outdoor thermal conditions. However, solely relying on rational indexes is insufficient in providing full ranges of contextual and personal determinants on human comfort. The underpinning assumption of rational indices, which equates the heat balance between human body and surroundings with thermal comfort, was often violated and might give inconsistent results in empirical investigation. Great discrepancies were found between the calculated index value and the actual thermal sensations voted by the individual subjects [7]. Besides a variety of microclimatic factors regarding thermal condition in outdoor environment, individuals’ perceptions of the environmental stimuli based on individual differences and features, and psychological and behavioral factors also contribute to the holistic assessment of subjective comfort in outdoor public spaces [11,25,26,27,28].

As defined by ASHRAE, thermal comfort is that “state of mind in which human feels satisfied within the thermal environment” [29]. The salient evolution of comfort model regarding psychological adaptation has been created referring to perception of and reaction to sensory information due to individuals’ experiences and expectations in a certain context [20]. The active role of human agent has been revealed and recognized, which underpins the adaptive approach for thermal comfort modeling with consideration of human adaptations in terms of physiological, behavioral, and psychological dimensions [30,31,32]. Further, the comprehensive conceptual model has been developed with expanded scope of influential factors, that indicates the ever-increasing importance of the personal physiological, psychological, and behavioral variables as well as non-thermal contextual factors [25,27,33].

The outdoor comfort is rather difficult to be measured in physical or psychological quantities, thus, it is generally conceptualized as a latent construct in this study. The biometeorological index, as well as the individuals’ socio-demographical characteristics, long-established attitude towards urban public spaces, and momentary general environmental perception on the overall environment are needed to be synthesized to predict comfort in outdoor urban environment [25,33,34,35,36]. By reviewing the conceptual models [27,33], we have included some influential factors for conceptualizing individuals’ momentary general environmental perception in urban public spaces, which may result in certain psychological effects on the holistic comfort. Moreover, the general environmental perception and the holistic comfort assessment are both hypothetically affected by individuals’ long-established attitudes towards urban public spaces, which reflects individuals’ experiences and socio-demographical backgrounds. To achieve a more comprehensive and robust model, an expanded set of factors are taken into account in our conceptual framework of comfort assessment. We address the new conceptual framework and conclude this study by proposing an approach to integrate individuals’ socio-demographical characteristics, long-established attitudes towards urban public spaces, momentary general environmental perceptions and rational thermal index into comfort modeling.

## 2. Conceptual Framework

The interplay between urban environmental condition and inhabitants’ quality of life makes the urban system as a whole. In turn, the investigations into outdoor comfort in urban public open spaces need combined and interdisciplinary approaches for gaining a holistic perspective. Individuals’ comfort perception is influenced by microclimatic and environmental stimuli to varying degrees based on psychological and behavioral adaptation, which is biased by their socio-demographical characteristics and living and working conditions. People with different demographic characteristics and socio-economic status are likely to experience different environments regarding certain behavioral patterns [37]. Therefore, microclimatic, and environmental conditions need to be linked to individuals’ personal perceptions based on their different social and behavioral factors. The proposed conceptual framework emphasizes on the impacts from the long-established attitudes towards urban public spaces and momentary general environmental perceptions on the holistic comfort assessment in urban public spaces. The underlying assumption is that individuals’ attitudes and perceptions are determined by their social background and experience. As already presented in the previous conceptual model, both long-term and short-term components are effectual in comfort assessment [27].

The measurements of comfort are not geographically or spatially invariable, which implies the importance of momentary perceptions regarding environmental features, and the contextual attributes regarding socio-cultural and climatological backgrounds. Many empirical evidences indicate the effects of the seasonal, geographical, and cultural differences on people’s thermal adaption in terms of physiological, psychological, and behavioral aspects [38,39,40,41]. During the outdoor activities, people gather multisensory experiences that inform their state of comfort in public realm [42]. When applying an instrument of comfort assessment in different urban places, a key concern is to ensure if the measurement of the relevant constructs is spatially invariant, however, it is normally a failure because of the varying influences of individual’s environment perceptions [43,44]. The difference of climate responsive design strategies regarding urban geometry, planting vegetation, cool surface and water bodies in various urban open spaces has been addressed to improve the outdoor thermal comfort conditions [5]. Apart from that, some spatial features of places or spots within a certain public space may have psychological effects on the occupants’ comfort assessment in different ways [33,34,45]. From this point of view, we speculate the individual’s general perceptions on place-related differences may cause the non-independence of comfort assessment. From the literature, few studies systematically delve into this issue regarding environmental attitudes and perceptions.

**Figure 1:**
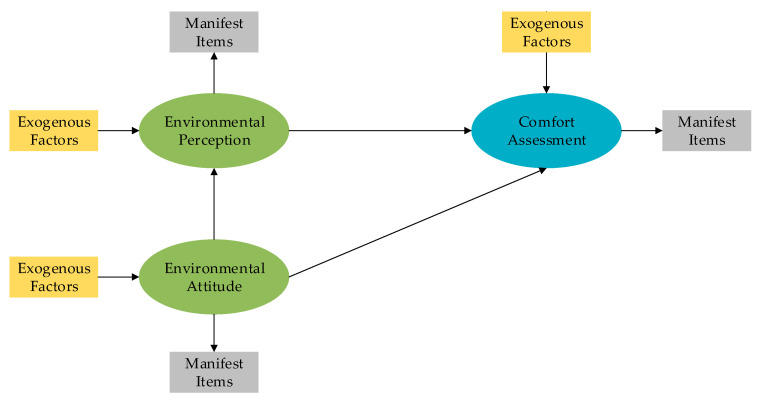
The diagram of conceptual framework.

The hypothetical conceptual framework is depicted in Figure 1. In this diagram, the oral components denote latent variables regarding individuals’ attitudes towards urban public spaces, momentary general environmental perceptions and comfort assessment, which are constructed by indicators measured through the questionnaire. The exogenous variables regarding individuals’ socio-demographic characteristics are assumed to impact on these three latent variables. The thermal index calculated by measured meteorological variables hypothetically influences individuals’ outdoor comfort assessment as well. As shown in the diagram, the relationships among latent variables are proposed, which indicates that individuals’ momentary general environmental perceptions may influence comfort assessment. In addition, the long-established attitudes towards urban public spaces may impact on momentary general environmental perceptions and comfort assessment.

The diagram comes up with the detailed hypotheses regarding (I) the relationships between latent variables and manifest items, and (II) the relationships between exogenous factors and latent variables, which are listed as follow.

(I). hypothetical relationships between latent variables and manifest items:

**Hypothesis** **1a (H1a).** *Environmental attitude is measured by the answer to question “Do you agree that public green space is the most important infrastructure?”.*

**Hypothesis** **1b (H1b).** *Environmental attitude is measured by the answer to question “Do you agree that public green space is conductive to spirit restoration and relaxation?”.*

**Hypothesis** **1c (H1c).** *Environmental attitude is measured by the answer to question “Do you agree that open space is necessary in both residential neighborhoods and business districts?”.*

**Hypothesis** **1c (H1d).** *Environmental attitude is measured by the answer to question “Do you agree that you prefer outdoor activities to indoor activities?”.*

**Hypothesis** **1e (H1e).** *Environmental attitude is measured by the answer to question “Do you agree that people should spend more time for outdoor activities?”.*

**Hypothesis** **1f (H1f).** *Environmental attitude is measured by the answer to question “Do you agree that recent weather is conductive to outdoor activities?”.*

**Hypothesis** **1g (H1g).** *Environmental attitude is measured by the answer to question “Do you agree that more investments are needed to manage and maintain the urban public spaces?”.*

**Hypothesis** **2a (H2a).** *Environmental perception is measured by the perception on green space in study area.*

**Hypothesis** **2b (H2b).** *Environmental perception is measured by the perception on facilities in study area.*

**Hypothesis** **2c (H2c).** *Environmental perception is measured by the perception on barrier-free design in study area.*

**Hypothesis** **2d (H2d).** *Environmental perception is measured by the perception on hygienic condition of study area.*

**Hypothesis** **2e (H2e).** *Environmental perception is measured by the perception on openness of study area.*

**Hypothesis** **2f (H2f).** *Environmental perception is measured by sensation of noise in study area.*

**Hypothesis** **2g (H2g).** *Environmental perception is measured by sensation of air quality in study area.*

**Hypothesis** **3a (H3a).** *Comfort assessment is measured by thermal sensation in study area.*

**Hypothesis** **3b (H3b).** *Comfort assessment is measured by the sensation of humidity in study area.*

**Hypothesis** **3c (H3c).** *Comfort assessment is measured by the sensation of wind in study area.*

**Hypothesis** **3d (H3d).** *Comfort assessment is measured by the sensation of radiation in study area.*

**Hypothesis** **3e (H3e).** *Comfort assessment is measured by the sensation of sunlight in study area.*

(II). hypothetical relationships between exogenous variables and latent variables:

**Hypothesis** **4 (H4).** *The effect of age on Environmental Attitude.*

**Hypothesis** **5 (H5).** *The effect of gender on Environmental Attitude.*

**Hypothesis** **6 (H6).** *The effect of education level on environmental attitude.*

**Hypothesis** **7 (H7).** *The effect of income on environmental attitude.*

**Hypothesis** **8 (H8).** *The effect of age on momentary environmental perception.*

**Hypothesis** **9 (H9).** *The effect of gender on momentary environmental perception.*

**Hypothesis** **10 (H10).** *The effect of education level on momentary environmental perception.*

**Hypothesis** **11 (H11).** *The effect of income on momentary environmental perception.*

**Hypothesis** **12 (H12).** *The effect of visiting frequency on momentary environmental perception.*

**Hypothesis** **13 (H13).** *The effect of age on comfort assessment.*

**Hypothesis** **14 (H14).** *The effect of gender on comfort assessment.*

**Hypothesis** **15 (H15).** *The effect of education level on comfort assessment.*

**Hypothesis** **16 (H16).** *The effect of income on comfort assessment.*

**Hypothesis** **17 (H17).** *The effect of visiting frequency on comfort assessment.*

**Hypothesis** **18 (H18).** *The effect of PET on comfort assessment.*

**Hypothesis** **19 (H19).** *The effect of environmental attitude on momentary general environmental perception.*

**Hypothesis** **20 (H20).** *The effect of environmental attitude on comfort assessment.*

**Hypothesis** **21 (H21).** *The effect of momentary general environmental perception on comfort assessment.*

## 3. Methodology

### 3.1. Structural Equation Modeling (SEM)

Structural equation modeling has become an important analysis method for multivariate data in empirical research, which is recognizable with the typical multiple equations in the model [46]. Quantifying the outdoor comfort assessment is aimed at capturing the relationships among a set of influential factors derived from meteorological monitoring and questionnaire-based survey that contain measurement errors. Therefore, SEM are equipped to handle multiple measures of concepts and measurement error in this study.

The variables in SEM are divided into measured and latent variables. Measured variables are those directly derived from measurements and surveys, while latent variables refer to the information which is not measurable directly. As depicted in the conceptual framework, comfort assessment, long-established attitudes towards urban public spaces and momentary general environmental perceptions are constructed as three latent variables by relevant indicators in SEM.

SEM is conventionally specified with measurement model and structural model. The measurement model defining the hypothesized relationships between latent variables and indicators (manifest items) can be expressed as follows [47]

(1) $$y_{i}=\alpha_{y}+\Lambda_{y}\eta_{i}+\varepsilon_{i}$$

(2) $$x_{i}=\alpha_{x}+\Lambda_{x}\gamma_{i}+\zeta_{i}$$

where $y_{i}$ is a vector of ordinal manifest items for a vector of endogenous latent variable $\eta_{i}$. $x_{i}$ is a vector of ordinal manifest item for a vector of exogenous latent variable $\gamma_{i}$. $\alpha_{y}$ and $\alpha_{x}$ are intercept vectors for indicator vectors of $y_{i}$ and $x_{i}$ respectively. $\Lambda_{y}$ is a matrix of factor loadings (coefficients) giving the effects of $\eta_{i}$ on $y_{i}$, $\Lambda_{x}$ is a matrix of factor loadings (coefficients) giving the effects of $\gamma_{i}$ on $x_{i}$. $\eta_{i}$ is a vector of endogenous latent variables. $\gamma_{i}$ is a vector of exogenous latent variables with $E\left(\gamma_{i}\right)=K$ and $Cov\left(\gamma_{i}\right)=\Phi$ (a variance–covariance matrix of latent variables $\gamma_{i}$). $\varepsilon_{i}$ denotes a vector of unique error component with $E\left(\varepsilon_{i}\right)=0$ and $Var\left(\varepsilon_{i}\right)=\Theta_{\varepsilon }$ (a matrix residual variances for $y_{i}$, assuming measurement errors $\varepsilon_{i}$ are uncorrelated with all other measurement errors and latent variables $\eta_{i}$), $\zeta_{i}$ is a vector of measurement errors in $x_{i}$ with $E\left(\zeta_{i}\right)=0$ and $Var\left(\zeta_{i}\right)=\Theta_{\zeta }$ (a matrix residual variances for $x_{i}$, assuming measurement errors $\varepsilon_{i}$ are uncorrelated with all other measurement errors and latent variables $\gamma_{i}$). In addition, $\varepsilon_{i}$ is assumed uncorrelated with $\zeta_{i}$.

The structural model accounts for the relationships among a set of variables simultaneously, which is defined as

(3) $$\eta_{i}=\alpha_{\eta }+B\eta_{i}+\Gamma \gamma_{i}+\varphi_{i}$$

where $\alpha_{\eta }$ is a vector of intercepts, B is the matrix of structural coefficients for the effects of among $\eta_{i}$ (assuming |I−Β|≠0), Γ is the structural coefficient matrix between $\eta_{i}$ and $\gamma_{i}$, $\varphi_{i}$ is a vector of error terms in $\eta_{i}$ with $E\left(\varphi_{i}\right)=0$ and $Var\left(\varphi_{i}\right)=\Psi$ (a diagonal matrix of residual variances for $\eta_{i}$, assuming the error terms $\varphi_{i}$ are uncorrelated with all other errors terms and latent variables $\gamma_{i}$). It follows as

(4) $$E\left(\eta_{i}\right)={\left(I-Β\right)}^{-1}\left(\alpha_{\eta }+\Gamma K\right)$$

and

(5) $$Cov\left(\eta_{i}\right)={\left(I-Β\right)}^{-1}\left(\Gamma \Phi \Gamma^{\prime }+\Psi \right){\left(I-Β\right)}^{-1}.$$

The mean structure for the measured variables of a general structural equation model parameterized in Ω, which denotes the vector of model parameters and can be written as

(6) $$\mu \left(\Omega \right)=\left[\begin{array}{c} \mu_{y}\\ \mu_{x} \end{array}\right]$$

where $\mu_{y}=\alpha_{y}+\Lambda_{y}{\left(I-Β\right)}^{-1}\left(\alpha_{\eta }+\Gamma K\right)$ and $\mu_{x}=\alpha_{x}+\Lambda_{x}K$.

The covariance structure Σ(Ω) can be expressed as

(7) $$\Sigma \left(\Omega \right)=\left[\begin{array}{cc} \Sigma_{yy} & \Sigma_{yx}\\ \Sigma_{xy} & \Sigma_{xx} \end{array}\right]$$

where $\Sigma_{xx}=\Lambda_{x}{{\Phi \Lambda }^{\prime }}_{x}+\Theta_{\zeta }$, $\Sigma_{yy}=\Lambda_{y}{\left(I-Β\right)}^{-1}\left(\Gamma \Phi \Gamma^{\prime }+\Psi \right){\left(I-Β\right)}^{-1}{\Lambda^{\prime }}_{y}+\Theta_{\varepsilon }$, and $\Sigma_{xy}=\Lambda_{y}{\left(I-Β\right)}^{-1}{{\Gamma \Phi \Lambda }^{\prime }}_{x}$.

For the ordinal measured variables, the variances of measurement errors can be identified by either standardizing the measured indicators or the measurement errors. Therefore, $\Theta_{\zeta }$ and $\Theta_{\varepsilon }$ are constrained as

(8) $$\Theta_{\zeta }=I-\mathrm{diag}\left(\Lambda_{x}{{\Phi \Lambda }^{\prime }}_{x}\right)$$

(9) $$\Theta_{\varepsilon }=I-\mathrm{diag}\left(\Lambda_{y}{\left(I-Β\right)}^{-1}\left(\Gamma \Phi \Gamma^{\prime }+\Psi \right){\left(I-Β\right)}^{-1}{\Lambda^{\prime }}_{y}\right).$$

As the consequence, the relationships between latent variables and measured indicators are estimated via analysis of Σ(Ω) using the ordinal data.

The SEM estimate were conducted based on the estimator of robust weighted least squares (WLSMV). WLSMV is the optimal solution for modeling categorical or ordered data, no assumptions of normally distributed variables are needed. Multiple Indicators Multiple Causes (MIMIC) modeling was applied to estimate the effects of covariates on the hypothetical latent factors.

### 3.2. Data Collection

#### 3.2.1. Study Sites

**Figure 2:**
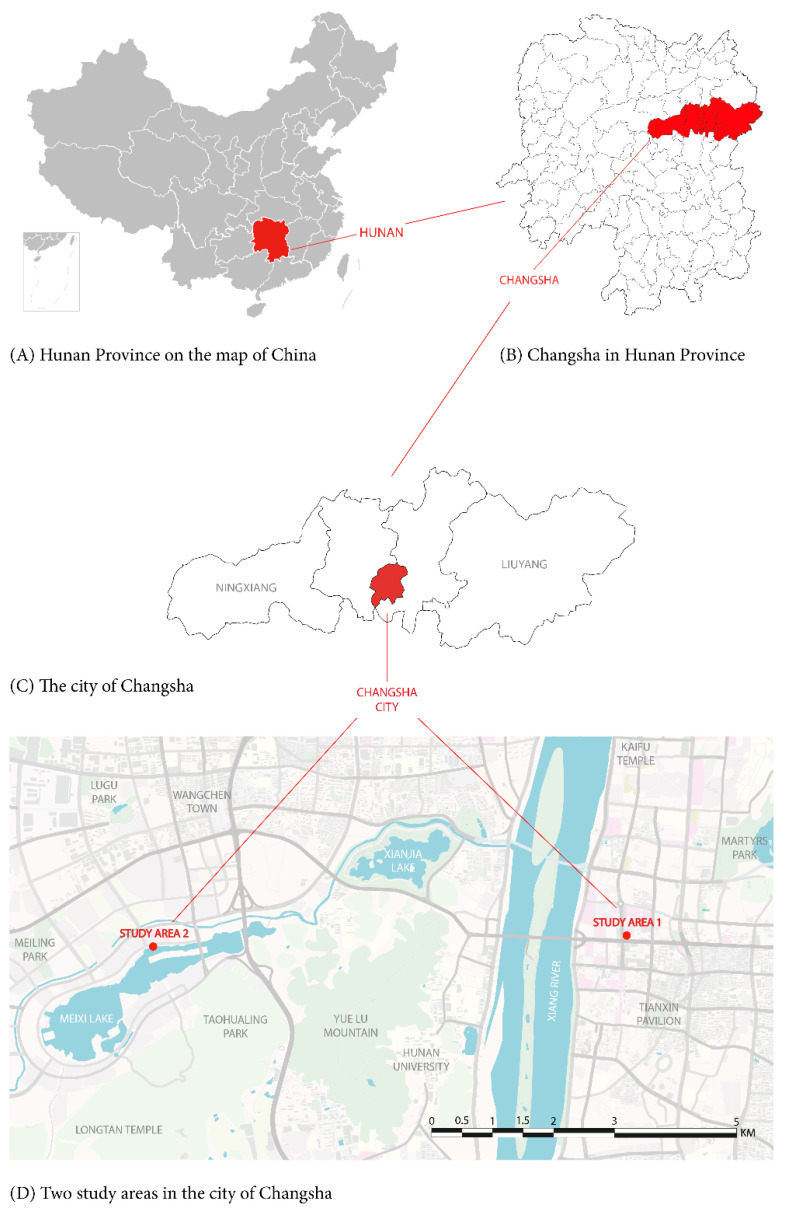
Locations of study areas.

The data used in this study come from field investigations carried out at the beginning of November 2019 in Changsha, China. The study location is depicted in Figure 2. Changsha is the capital and the largest city in Hunan province in the central south of China, where more than 3 million population lives in. Since located in the humid subtropical climate zone (Cfa) based on the Köppen–Geiger climatic classification [48], Changsha city is featured with four distinct seasons. It is the cool Autumn of Changsha when the outdoor surveys and measurements of this study were conducted. The dataset contains questionnaire-based surveys in two different public squares located in the inner and fringe city. Both study areas are designed for providing the open space for inhabitants and visitors.

Wuyi square (study area 1) is the traditional public space in the central commercial area of the downtown in Changsha. It is known as a landmark with many shopping malls, bar street and the famous walking commercial street located nearby, as well as a central hub of two subway lines with hundreds of thousands of people transferring in the station by the name of Wuyi square. The whole square comprises of green spaces, pavements, paved small squares and sunken spaces. The south and west sides of Wuyi square are next to the main roads with heavy traffic and pedestrian flows. In the fast-paced city center, Wuyi square consistently provides the space for rest and relaxation. Meixihu park (study area 2) is located in the peri-urban area, where an artificial lake is surrounded by pedestrian-only pathways, and green spaces and squares with different themes and sizes. Lots of spaces for rest, leisure, and physical exercise, as well as great views of beautiful natural sceneries are provided in Meixihu park. Many residents and tourists visit these two public spaces when the weather is suitable for outdoor activities.

#### 3.2.2. Field Measurement and Survey

The data collection was conducted in two study areas by research assistants from 2 November 2019 to 9 November 2019, which consists of measurements of meteorological variables and questionnaire-based surveys. Since the weather in November is mild and with little rain, the impacts of psychological factors related to adaptation may be more effective and easier to identify. The monitoring devices was deployed and tested before fieldwork, complying with ISO 7726 [49]. All sensors of microclimatic variables were mounted on a movable tripod, which were used for monitoring the air temperature ($T_{a}$), relative humidity (RH), global temperature ($T_{g}$), and wind velocity (v). The specification of sensors is listed in Table 1. $T_{g}$ was measured by a black globe thermometer with 150 mm diameter. The measurement height was set at 1.1 m according to the average height of the gravity center of adults. All meteorological data were recorded automatically by the data logger.

The questionnaire form is in Chinese, which is comprised of 3 sections of questions respectively about respondents’ (1) socio-demographical characteristics, (2) attitudes toward urban public spaces, and (3) momentary environmental perceptions regarding green spaces, facilities, hygienic conditions, barrier-free design, openness, noise and air quality, and thermal sensation, and sensations of humidity, wind, radiation, and sunlight. In the second part of attitude questions, each respondents’ statement was measured with five-point Likert scales with 1 = strongly disagree to 5 = strongly agree. Perceptions on various environmental attributes were measured in the third part of the form with seven-point scale from −3 to 3 to denotes the different levels of perceptions to different attributes. As for comfort assessment, the seven-point scale from −3 to 3 is also used to denote the levels from “very discomfort” to “very comfort”. Research assistants randomly invited people in the study areas to participate in the questionnaire-based survey. If they agreed to join, the survey was carried out with an explanation of study intention. Each survey took around 10 min. The beginning and ending time were recorded, which is used to correspond each questionnaire with the simultaneous measurement of meteorological variables. After four inconsecutive days’ field work, 372 valid questionnaires were collected and saved.

## 4. Result and Discussion

### 4.1. Descriptive Statistics

The mean radiant temperature ($T_{mrt}$) is a synthetic variable with primary importance in the studies of thermal sensation. All long-wave and short-wave radiation are combined by $T_{mrt}$, that is defined as the uniform temperature of an ideal enclosure in which the radiant heat transfer from human body is equal to the radiant heat transfer in the actual non-uniform enclosure. $T_{mrt}$ is calculated according to ISO 7726 [49] as:

(10) $$T_{mrt}={\left[{\left(T_{g}+273\right)}^{4}+\frac{1.10\times 10^{8}\times v^{0.6}\left(T_{g}-T_{a}\right)}{\xi D^{0.4}}\right]}^{0.25}-273$$

where $T_{g}$ is global temperature, $T_{a}$ is air temperature, v is wind velocity, D is the diameter (=150 mm) of black ball sensor for $T_{g}$ and ξ is the emissivity coefficient (=0.95).

PET represents the integral impact of meteorological variables on thermal sensation in outdoor environments. Derived from heat balance model (Munich energy-balance model for individual, MEMI), PET has been widely used in comfort studies in different climate zones [38]. In this study, PET is calculated by using RayMan [50,51]. According to the observations, no significant difference between clothing and activity level was found. The calculation assumes the 80 w for activity level and 0.9 clo for clothing level of an average person.

The meteorological variables and PET in study areas during the survey period are illustrated in Table 2. The large range of variations regarding the measured $T_{a}$ and $T_{g}$ is in accordance with the typical local autumn climate in Changsha. The relative humidity during the survey period is comparatively low based on the local annual climate, since November is one of the driest months in Changsha. The mean value of wind velocity is 0.56 m/s, and the standard deviation is relatively small.

The statistical information regarding socio-demographical characteristics of respondents is shown in Table 3. It indicates a bit more females than males participated and completed the surveys. The majority of respondents are under the age of 40 years, in contrast, only 1.9% of respondents are the elderly. Perhaps the main reason centers on the location of survey. Wuyi square is located in the commercial area which is a preferred gathering place of young people. Meixihu park is situated in the urban district of many universities and surrounded by newly developed dwelling communities, most visitors in this area are students from nearby universities, young couples, and new residents migrating from other cities and villages. Most respondents’ figure fits the normal level of BMI, but there are still 15.3% of respondents with a risk of obesity [52]. Almost four fifth respondents are unmarried, including singles and divorcees. Regarding the education level, about 79.8% of respondents have college-trained background. The percentage of unemployed respondents is much higher than employed ones, since the unemployed respondents comprise of the jobless, freelancer, self-employed persons and students. In addition, there are six out of ten respondents making less than 5000 CNY per month.

The data regarding behavioral variables of respondents are collated and listed in Table 4. As we discovered, the top highest proportion of respondents’ visiting purposes are “taking a walk” (40.6%) and “waiting for commute” (31.7%). On the contrary, less than 2% of respondents came to the study areas for physical exercise. As for the transportation mode, more than half of respondents went to the study areas by bus and metro, since both study areas are very close to metro stations and bus stops. Up to 70% of respondents spent more than 1 h totally in outdoor environments before the survey. About 25% of respondents stayed in the study areas for more than 1 h before the survey. Less than 20% of respondents visited the study areas for the first time. Besides, the majority of respondents visited the study areas less than once per week.

### 4.2. Results of SEM 

**Figure 3:**
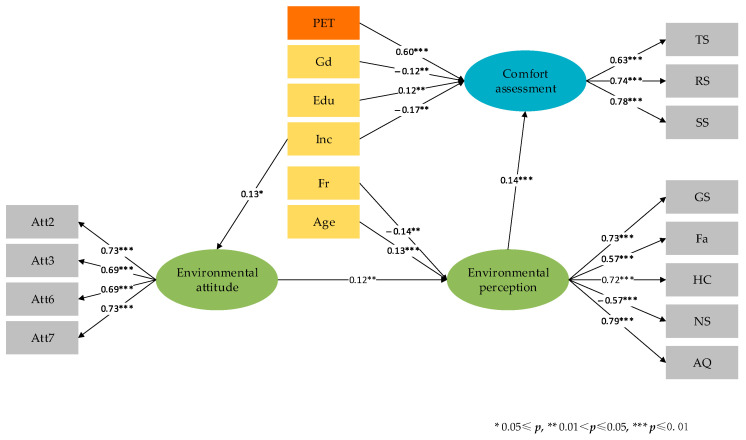
Diagram of SEM regarding comfort assessment in urban public spaces.

The estimation results regarding verification of hypothetical relationships are listed in Table 5. According to the indices of model fit shown in Table 6, the model in this study has a good fit identified by the Comparative Fit Index (CFI = 0.94), Tucker Lewis Index (TLI = 0.93), and Root Mean Square Error of Approximation (RMSEA = 0.05) [53]. The diagram of SEM illustrates the significant connections in measurement model and structural model (see Figure 3). Meanwhile, the detailed results of SEM estimate are shown in Table 7.

Regarding the components of measurement model in SEM, the hypothetical relationships between the latent variables and corresponding indicators are identified. The respondents’ attitudes towards urban public spaces are significantly measured by indicators of Att2, Att3, Att6, and Att7. The rest of related questions in surveys are not feasible to measure respondents’ long-established attitudes towards urban public spaces. As for respondents’ momentary general environmental perception, the perceptions on green spaces, facilities and hygienic conditions, and the sensations of noise and air quality in study areas are effective indicators. The holistic comfort assessment can be measured by thermal sensation, sensations of surrounding radiation and sunlight. However, the relationships between comfort assessment and sensations of humidity and wind are not verified as significant.

Based on the estimate results for structural relationships, the links between latent factors and some socio-demographical characteristics are significant. The income positively influences the long-established attitudes towards urban public spaces. It is widely believed that people with high-income levels potentially pay more attention to the quality of outdoor environments and participate more outdoor activities for health outcomes. The direct effect of respondents’ income on their comfort assessment is negative, which indicates that people of high-income level are reluctant to adapt to be comfortable in the same outdoor environment. The respondents’ age positively impacts on their momentary general environmental perceptions, which means older people are inclined to have more positive environmental perceptions in the same setting and conditions in urban public spaces. As for behavioral factors, the visiting frequency of respondents is negatively proportional to their momentary general environmental perception. Further, since the momentary general environmental perception in the study areas positively affects comfort assessment, people visited the study areas less felt more comfortable during the surveys. Gender difference was found that female respondents are more likely to feel comfortable during surveys. Regarding the education, respondents with higher education are apt to give higher comfort assessment. As for the physical thermal condition, the PET was calculated which positively influences comfort assessment in the study areas. This is perhaps because the surveys were conducted in November with relatively cold temperature in outdoor environments in Changsha.

The comfort assessment was measured by direct questions, or simply treated as thermal sensation in previous empirical investigations. Thus, it was hard to understand the comfort perceptions from multifarious perspectives. As shown in the results, through the SEM approach, a latent construct of comfort is conceptualized, which has been measured by indicators from different perspectives. Although, the relationships between outdoor comfort assessment and individuals’ socio-demographical, psychological, and behavioral factors were discussed in some of previous studies [25,27,35,45,54], the indirect effect of long-term established attitudes on comfort assessment is first verified in this study. This implies the assessment of comfort depends on perceptions based on momentary place-related and person-related conditions, and longstanding subjective attitudes based on experience, knowledge, etc.

## 5. Conclusions

This study presents a comprehensive conceptual model regarding the relationships between outdoor comfort and individuals’ long-established attitudes towards urban public spaces and momentary general environmental perceptions. A structural equation model was estimated using the data of 372 subjects surveyed in two public spaces in Changsha city. Most of the hypothetical relationships proposed in the conceptual framework are verified. Unlike previous studies, individual’s holistic comfort assessment is conceptualized as a latent variable, which is unmeasurable and can only be measured by the indicators regarding thermal sensation, and sensations of radiation and sunlight during the surveys in study areas. The sensations of wind and humidity measured in the surveys are not significantly correlated with comfort assessments in the local context as presented in the results. Nevertheless, as a latent construct, individual’s holistic comfort assessment is expected to be measured by specific sensations in different context of geographical regions.

The results of SEM estimate provide quantitative evidence, which indicates physical thermal exposure condition is the strong effect on individual’s comfort assessment. Meanwhile, the important role of person-related variables in outdoor comfort modeling has been revealed. The mechanism of comfort perception involves the long-established attitude towards urban public spaces and the momentary general environmental perception in accordance with the previous conceptual model of comfort perception proposed by Lenzholzer and de Vrijs (2019) [27]. Individual’s comfort in urban public spaces is not only based on the current state when the comfort perception is recorded but also the attitudes established in the outdoor experience and socio-demographical factors.

Unlike previous empirical investigations focusing on the momentary influential factors only, this study emphasizes on the importance of individual’s socio-demographical characteristics and long-term established psychological factors in outdoor comfort modeling. More empirical evidence related to the respondents with various socio-demographical backgrounds in different geographical regions are expected to be carried out in the coming future.

## Figures and Tables

**Table 1:** The specification of sensors for assembly movable microclimate monitor.

Variable	Sensor Model	Range	Accuracy
*T_a_*	S-THB-M002	−40–75 °C	±0.2 K
*T_g_*	SPA 150	−50–250 °C	±0.3 K
*RH*	S-THB-M002	0–100%	±3%
*v*	S-WSET-A	0–45 m/s	±1.1 m/s

**Table 2:** Meteorological variables and thermal index in field studies.

Variable	Mean	Median	Minimum	Maximum	SD
*T_a_* (°C)	25.8	25.6	17.6	35.4	3.7
*RH* (%)	39.4	36.0	22.4	67.4	11.4
*v* (m/s)	0.56	0.53	0.16	1.67	0.28
*T_g_* (°C)	29.3	29.3	16.9	41.5	6.4
*T_mrt_* (°C)	35.0	30.0	16.2	62.1	13.2
PET (°C)	26.3	26.6	16.3	36.1	4.8

**Table 3:** Socio-demographics of respondents in surveys.

Variable	Class Condition	Percentage
Gender	Male	41.9%
	Female	58.1%
Age	<20	42.2%
	20–39	49.5%
	40–59	6.5%
	≥60	1.9%
BMI	<18.5	20.2%
	18.5–24	64.5%
	≥24	15.3%
Civil status	Married	21.2%
	Unmarried	78.8%
Education	High school or below	14.8%
	Graduate degree	79.8%
	Postgraduate degree	5.4%
Employment	Employed	42.2%
	Unemployed and others	57.8%
Monthly income	<5000 CNY	66.7%
	5000–10,000 CNY	24.2%
	≥10,000 CNY	9.1%

**Table 4:** Behavioral factors of respondents in surveys.

Variable	Class Condition	Percentage
Purpose	Taking a walk	40.6%
	Social activity	9.7%
	Rest	8.3%
	Waiting for commute	31.7%
	Physical exercise	1.9%
	Others	7.8%
Transportation mode	Walking	18.8%
	Bike	5.1%
	Bus and metro	55.4%
	Taxi or online hailing car	9.4%
	Private car	9.1%
	Others	2.2%
Total outdoor duration	<30 min	6.2%
	30–60 min	24.5%
	60–90 min	23.7%
	90–120 min	18.0%
	≥120 min	27.7%
Duration in study area	<15 min	17.5%
	15–30 min	25.8%
	30–45 min	19.4%
	45–60 min	12.4%
	≥60 min	25.0%
Frequency of visiting	First time	19.9%
	Scarcely	30.6%
	Occasionally	35.5%
	Sometimes	10.5%
	Often	3.5%

**Table 5:** Verification result of hypothetical relationships.

Hypothesis	Related Variables	Estimate
H1a	Environmetal attitude and Att1	Invalid
H1b	Environmetal attitude and Att2	Valid
H1c	Environmetal attitude and Att3	Valid
H1d	Environmetal attitude and Att4	Invalid
H1e	Environmetal attitude and Att5	Invalid
H1f	Environmetal attitude and Att6	Valid
H1g	Environmetal attitude and Att7	Valid
H2a	Environmental perception and GS	Valid
H2b	Environmental perception and Fa	Valid
H2c	Environmental perception and BD	Invalid
H2d	Environmental perception and HC	Valid
H2e	Environmental perception and OP	Invalid
H2f	Environmental perception and NS	Valid
H2g	Environmental perception and AQ	Valid
H3a	Comfort assessment and TS	Valid
H3b	Comfort assessment and HS	Invalid
H3c	Comfort assessment and WS	Invalid
H3d	Comfort assessment and RS	Valid
H3e	Comfort assessment and SS	Valid
H4	Age and Environmental attitude	Invalid
H5	Gd and Environmental attitude	Invalid
H6	Edu and Environmental attitude	Invalid
H7	Inc and Environmental attitude	Valid
H8	Age and Environmental perception	Valid
H9	Gd and Environmental perception	Invalid
H10	Edu and Environmental perception	Invalid
H11	Inc and Environmental perception	Invalid
H12	Fr and Environmental perception	Valid
H13	Age and Comfort assessment	Invalid
H14	Gd and Comfort assessment	Valid
H15	Edu and Comfort assessment	Valid
H16	Inc and Comfort assessment	Valid
H17	Fr and Comfort assessment	Invalid
H18	PET and Comfort assessment	Valid
H19	Environmental attitude and Environmental perception	Valid
H20	Environmental attitude and Comfort assessment	Invalid
H21	Environmental perception and Comfort assessment	Valid

**Table 6:** Fit indices of SEM.

Criterion	CFI	TLI	RMSEA
Value	0.947	0.938	0.045

**Table 7:** Estimate result of SEM.

**Measurement Model**		**Variable**	**λ**	**S.E.**	***p*-Value**
Environmental perception	→	GS	0.73 ***	0.029	0.000
	→	Fa	0.57 ***	0.037	0.000
	→	HC	0.72 ***	0.034	0.000
	→	NS	−0.57 ***	0.036	0.000
	→	AQ	0.79 ***	0.030	0.000
Comfort assessment	→	TS	0.63 ***	0.038	0.000
	→	RS	0.74 ***	0.034	0.000
	→	SS	0.78 ***	0.033	0.000
Attitude towards urban public spaces	→	Att2	0.73 ***	0.024	0.000
	→	Att3	0.69 ***	0.027	0.000
	→	Att6	0.69 ***	0.028	0.000
	→	Att7	0.73 ***	0.024	0.000
**Structure Model**			**β**	**S.E.**	***p*-Value**
Environmental perception	←	EA	0.12 **	0.053	0.025
	←	Age	0.13 ***	0.051	0.009
	←	Fr	−0.14 **	0.059	0.019
Attitude towards urban public spaces	←	Inc	0.13 *	0.068	0.053
Comfort assessment	←	EP	0.14 ***	0.047	0.003
	←	Sex	−0.12 **	0.056	0.028
	←	Edu	0.12 **	0.054	0.033
	←	Inc	−0.17 **	0.078	0.033
	←	PET	0.60 ***	0.041	0.000

* 0.05 ≤ *p*, ** 0.01 < *p* ≤ 0.05, *** *p* ≤ 0.01.

## Data Availability

The data are available on request from the corresponding author. The data are not publicly available due to privacy reasons.

## Nomenclature

**Att1**	Answer to “Do you agree that public green space is the most important infrastructure?”
**Att2**	Answer to “Do you agree that public green space is conductive to spirit restoration and relaxation?”
**Att3**	Answer to “Do you agree that open space is necessary in both residential neighborhoods and business districts?”
**Att4**	Answer to “Do you agree that you prefer outdoor activities to indoor activities?”
**Att5**	Answer to “Do you agree that people should spend more time for outdoor activities?”
**Att6**	Answer to “Do you agree that recent weather is conductive to outdoor activities?”
**Att7**	Answer to “Do you agree that more investments are needed to manage and maintain the public spaces?"
**GS**	Perception on green space in study area
**Fa**	Perception on facilities in study area
**BD**	Perception on barrier-free design in study area
**HC**	Perception on hygienic condition of study area
**OP**	Perception on openness of study area
**NS**	Sensation of noise in study area
**AQ**	Sensation of air quality in study area
**TS**	Thermal sensation in study area
**HS**	Sensation of humidity in study area
**WS**	Sensation of wind in study area
**RS**	Sensation of radiation in study area
**SS**	Sensation of sunlight in study area
**Age**	Age of respondent
**Gd**	Gender of respondent
**Edu**	Education level of respondent
**Inc**	Monthly income of respondent
**Fr**	Frequency of visiting the study area
**PET**	Physiological equivalent temperature
